# Photocatalytic Behaviour of Zinc Oxide Nanostructures on Surface Activation of Polymeric Fibres

**DOI:** 10.3390/polym13081227

**Published:** 2021-04-10

**Authors:** Muhammad Tayyab Noman, Nesrine Amor, Michal Petru, Aamir Mahmood, Pavel Kejzlar

**Affiliations:** 1 Department of Machinery Construction, Institute for Nanomaterials, Advanced Technologies and Innovation (CXI), Studentská 1402/2, 461 17 Liberec 1, Technical University of Liberec, 46117 Liberec, Czech Republic; nesrine.amor@tul.cz (N.A.); michal.petru@tul.cz (M.P.); 2 Department of Material Engineering, Faculty of Textile Engineering, Studentská 1402/2, 461 17 Liberec 1, Technical University of Liberec, 46117 Liberec, Czech Republic; aamir.mahmood@tul.cz; 3 Department of Material Science, Faculty of Mechanical Engineering, Studentská 1402/2, 461 17 Liberec 1, Technical University of Liberec, 46117 Liberec, Czech Republic; pavel.kejzlar@tul.cz

**Keywords:** nZnO, photocatalytic activity, polymeric fibres, cotton, stabilization

## Abstract

Zinc oxide (ZnO) in various nano forms (nanoparticles, nanorods, nanosheets, nanowires and nanoflowers) has received remarkable attention worldwide for its functional diversity in different fields i.e., paints, cosmetics, coatings, rubber and composites. The purpose of this article is to investigate the role of photocatalytic activity (role of photogenerated radical scavengers) of nano ZnO (nZnO) for the surface activation of polymeric natural fibres especially cotton and their combined effect in photocatalytic applications. Photocatalytic behaviour is a crucial property that enables nZnO as a potential and competitive candidate for commercial applications. The confirmed features of nZnO were characterised by different analytical tools, i.e., scanning electron microscopy (SEM), field emission SEM (FESEM) and elemental detection spectroscopy (EDX). These techniques confirm the size, morphology, structure, crystallinity, shape and dimensions of nZnO. The morphology and size play a crucial role in surface activation of polymeric fibres. In addition, synthesis methods, variables and some of the critical aspects of nZnO that significantly affect the photocatalytic activity are also discussed in detail. This paper delineates a vivid picture to new comers about the significance of nZnO in photocatalytic applications.

## 1. Introduction

The last two decades are eye witness to a prestigious revolution made by nano science, as researchers enlarge their circle for nanomaterials especially metal oxide-based nanomaterials, i.e., zinc oxide (ZnO). Nano ZnO (nZnO) composed of different forms of nanostructures i.e., nanoparticles, nanorods, nanowires, nanobelts, nanosheets and nanoflowers, has gained significant attention from researchers for the fabrication of sensors [1,2,3], medical devices [4,5,6,7], composites [8,9,10,11] and photocatalysts [12,13,14] for various applications [15,16,17,18]. nZnO is a fascinating material that possesses and reveals exceptional physicochemical properties when used in photocatalytic applications. As a semiconductor, ZnO has high thermal conductivity, high exciton binding energy (60 m eV), high electron mobility and wide band gap, i.e., 3.2–3.4 eV [19]. The potential of photocatalytic activity of nZnO expands its scope in biomedical [20], industrial [21,22], catalysis [23,24], coatings [25,26], sensors [27,28], textiles [29,30] and energy conversion devices, i.e., fuel and solar cells [31]. nZnO has also been used in personal (sunscreen) and beauty care (cosmetics) products due to excellent ultraviolet (UV) absorption properties. UV rays are considered as the primary cause of skin diseases, i.e., wrinkles, skin cancer, aging and sunburns. On the nanoscale, ZnO shows significantly high optical, electronic and antimicrobial properties and due to these properties—nZnO provides great protection against the breaking down of skin collagen and the skin regeneration mechanism. Therefore, the addition of nZnO in the formulation of cosmetics not only protects our skin from harmful rays (UV-B and UV-A) but also enhances the attractiveness of beauty care. The photocatalytic activity of nZnO and its effects on the morphology of fibrous surface is a topic of great interest that should be addressed in detail. The literature discussed above only explains the application of photocatalytic activity of nZnO in different fields. However, the role of radical scavengers produced during the process of photocatalysis and their typical effect on the morphology and surface topography of polymeric fibrous material is still an area of investigation for the explanation of enhanced photocatalytic activity. As well as the authors searched, there was no such literature available on this hot issue. Therefore, our investigation provides an up-to-date knowledge on the role of photogenerated charge carriers (radical scavengers) and their induced potential for enhanced photocatalytic activity of nZnO and their combined effect on surface topography of polymeric fibrous materials. This investigation delineates a gateway for newcomers and experienced researchers in their respective areas as a valuable reference. In order to achieve our objectives, this review paper discusses the latest literature under two categories, i.e., the development of nZnO coated textiles and the role of photocatalytic activity on surface properties.

Polymeric fibres (cotton, polyester, jute, polyamide, wool and polypropylene) have been widely used in many fields of life due to versatility in their different properties. Surface roughness, porosity and capillary imbibition action in porous media are some of the very important properties of polymeric fibrous materials from textiles and composites point of view. Xiao et al. reported the application of a fractal model for capillary flow through a single tortuous capillary with surface roughness in porous media. The derived model is tested against imbibition mass and imbibition height. The results revealed that both of these characteristics decreased with an increase in relative surface roughness. It was also observed that the equilibrium time for rough surfaces decreased with an increment in relative roughness [32].

**Figure 1:**
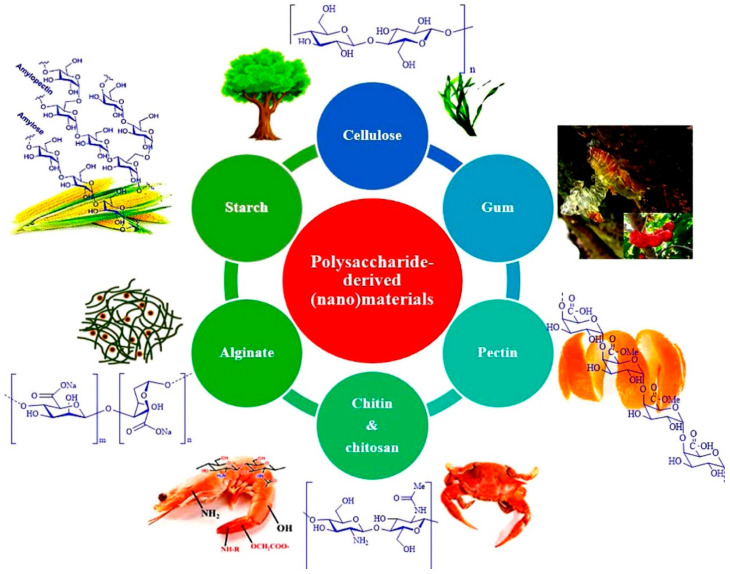
A group of sustainable and environmentally friendly polymeric carbohydrates (polysaccharides). Reprinted with permission from Reference [39]. Copyright 2021, with permission from Elsevier.

In a different study, Xiao et al. studied the fluid transport through fibrous porous media under a fractal model with dimensionless permeability and Kozeny–Charman constant. The obtained results were in good agreement with the previously performed studies and with the experimental data. The results explain that the physical phenomenon of fluids transport through porous media can be elucidated in a better way with this fractal model as the values of the Kozeny–Charman constant increases with an increase in porosity, tortuosity fractal dimension, relative roughness and pore dimensions [33]. Polysaccharides are a class of renewable and sustainable natural polymeric carbohydrates including chitosan, cellulose, gum, starch, alginate, pectin and chitin. The applications (energy storage devices, medical devices, composites, sorbents, nano catalysts and light weight porous materials) of these polymeric carbohydrates have increased tremendously during the last few years due to their biodegradability, non-toxicity, sustainability and environmentally friendly benefits [34,35,36,37]. In a recent study, Ahmed et al. discussed the benefits of chitosan and chitin in the fabrication of carbon-based composites for waste water treatment. They reported that due to mechanical properties and easy handling, these biopolymers not only show excellent compatibility for all kind of carbonaceous materials like carbon nanotubes, graphene, biochar, activated carbon and graphene oxide but also provide excellent results for the adsorption of water pollutants [38]. In a review article, Nasrollahzadeh et al. elucidated the role of bio polysaccharides, i.e., chitosan, pectin, cellulose and alginate in waste water treatment. Natural biopolymers are excellent candidates for the elimination of aqueous contaminants and aquatic pollution when utilised as nano sorbents or nano catalysts in the composition of nanobiocomposites. A list of the most used biopolymers or polymeric carbohydrates is presented in Figure 1 [39].

In our study, the range of polymeric fibres covers from cotton, jute, flax and their combinations with other fibres for a comparative analysis of photocatalytic activity of nZnO coated and noncoated samples. In a recent article, Theerthagiri et al. reviewed biological and energy applications (electrochemical supercapacitors, lithium-ion batteries, dye sensitised solar cells, photocatalyst, bioimaging, sensors, drug delivery, toxicity and gene delivery) of nZnO and explained that physicochemical properties are responsible for a dramatic change in nZnO behaviour and differentiates nZnO from its bulk counterpart. ZnO associated photocatalysis is a type of heterogeneous photocatalysis where the reactants, photocatalysts and the products are in different phases during the reaction mechanism. They discussed the effects of crystallinity, particle size and surface morphology on the performance efficiency of nZnO [40]. Nada et al. reported a successful allocation of Zn element in the polymer matrix of chitosan gelatine-based hybrid nanobiocomposites. Acid catalysed amino addition reaction was observed during the incorporation of zinc, inside the chitosan matrix and later on, the nanofibers of zinc incorporated chitosan were developed by an electrospinning process. The results elucidate that chitosan nanofibers with the addition of zinc element showed excellent antimicrobial properties [41]. However, it is still believed that our approach towards the critical aspects of photocatalytic performance of nZnO is exceptional and provides a vivid analysis for future studies. In addition, this review paper uncovers new horizons for researchers working with textiles and provides new ideas about the utilization of nZnO in photocatalytic applications.

**Figure 2:**
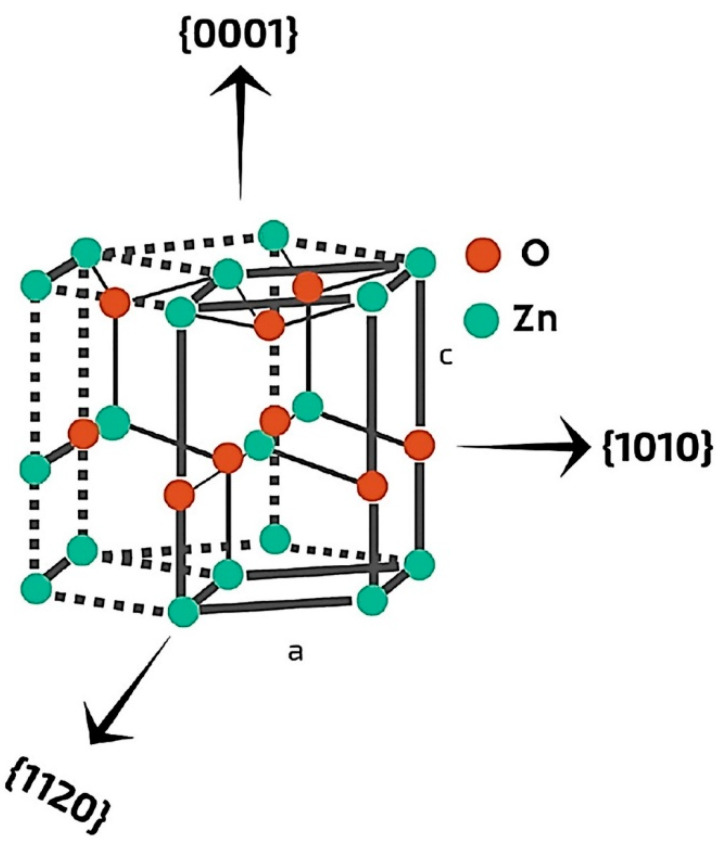
A typical wurtzite crystal structure of ZnO with indicated directions. Reprinted with permission from Reference [46]. Copyright 2017, with permission from Elsevier.

In recent years, plenty of articles have been published explaining the significance of nZnO as a photocatalyst [42]. However, there is still a vacant place to dig up for dimensions, synthesis routes and variation in size, that can explain the photocatalytic behaviour of nZnO in a better way. The recent literature explains the selection of a suitable synthesis route and that is very important to control the size and to harness maximum photocatalytic performance. Therefore, we started with synthesis routes and important variables involved during the synthesis, then discussed their critical exigency for better photocatalytic activity, elaborated on individual articles that provided new insights and ended up with photocatalytic applications of nZnO coated textiles. Synthesis routes and parameters elucidated a crucial impact on the dimensional stability of nZnO. In an experimental study, Abramova et al. developed medically proven antimicrobial textiles by coating sol-gel synthesised nZnO on the surface of fabric through ultrasonic irradiations. They explained that the fabricated samples coated with zinc oxide in combination with titanium dioxide exhibited 99.99% suppression level of Escherichia coli (*E. coli*). These results reveal that sol-gel is an excellent method to deposit nanostructures on the surface of textiles [43]. Barreto et al. used a microwave-assisted method for the fabrication of nZnO and found that selected variables and reagents, i.e., temperature, additives, time and microwave power impart significant impact on growth, morphology, shape and size of nZnO. They explained that the addition of surfactants enhances the photocatalytic performance of nZnO to a significant level [44]. In a recent study, Noman et al. synthesised ZnO nanoparticles (ZnO NPs) via sonication and coated them on cotton and polyester fabrics with varying thickness. The results reveal that a smooth nZnO coating on both fabrics significantly depends on ultrasonic irradiation’s time and intensity. Moreover, longer irradiation time enhances the porosity that allows nZnO to go deeper and creates a smooth layer on both fabrics. These results depict that a higher amount and a smooth coating of nZnO significantly enhance the photocatalytic performance of investigated samples [45]. It is observed from the previous literature that the structure of ZnO plays a crucial role in the augmentation of photocatalytic activity. ZnO exists in two main crystal structures, i.e., cubic blend and hexagonal wurtzite. However, hexagonal wurtzite is the most common and most stable form of ZnO at ambient conditions. Krol et al. explained the lattice parameters, i.e., a = 0.325 nm and c = 0.521 nm, with three growth directions of hexagonal wurtzite crystal where every tetrahedral Zn atom is surrounded by four O atoms. These parameters of the wurtzite structure are responsible for higher photocatalytic efficiency than the cubic zinc blend. A typical wurtzite crystal structure with primary growth directions is illustrated in Figure 2 [46].

**Figure 3:**
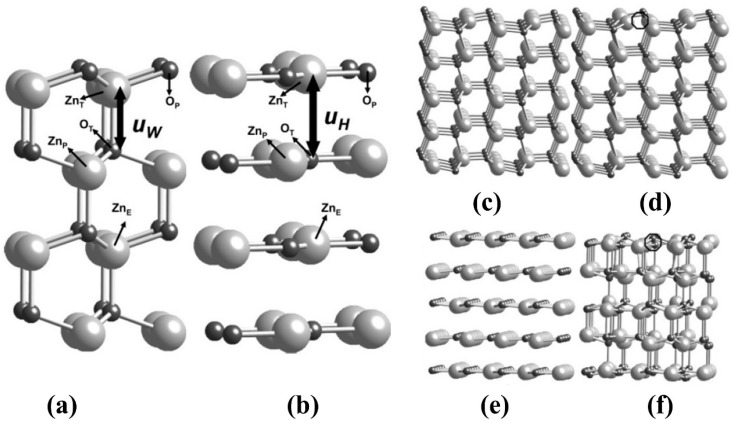
(**a**) Ball and stick model of ZnO with wurtzite structure. The light spheres denote Zn atoms while dark spheres denote O atoms. Zn and O atoms are in different planes (**b**) ZnO with hexagonal structure. Both O and Zn atoms are in the same plane (**c**) ZnO stoichiometric structure with five layers, (**d**) ZnO stoichiometric structure after removing O atom, (**e**) Single layer ZnO stoichiometric structure, and (**f**) Single layer ZnO stoichiometric structure after removing O atom. Reprinted with permission from Reference [47]. Copyright 2007, with permission from the American Chemical Society.

However, in a previous study, Pala and Metiu evaluated the density functions of ZnO-based thin films to calculate the amount of energy required during the formation of oxygen vacancy and structures of plain, hybrid and thin films used in pure and hybrid forms for photocatalytic applications. The results elucidate that after the removal of oxygen vacancy, the wurtzite crystal structure solely depends on the thickness of thin layers of ZnO as presented in Figure 3 [47].

**Figure 4:**
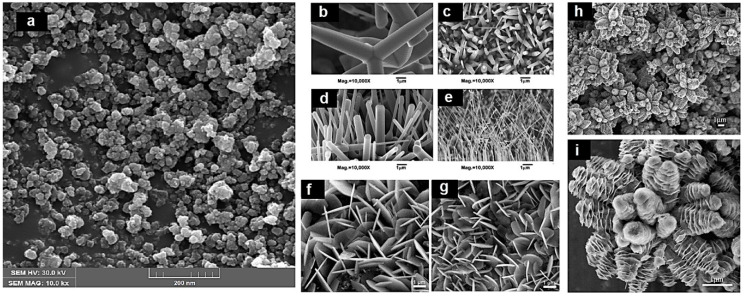
Scanning electron microscopy (SEM) and field emission SEM (FESEM) images for: (**a**) 0D (nanoparticles); (**b**–**e**) 1D (nanorods); (**f**,**g**) 2D (nanofilms); and (**h**,**i**) 3D (nanoflowers), respectively. Reprinted with permission from References [50,51,52,53]. Copyright 2009, 2013, and 2014, respectively, with permission from Elsevier.

Ong et al. thoroughly reviewed and explained the synthesis mechanism of nZnO and described the properties responsible for higher photocatalytic activity. They categorised ZnO as a green eco-friendly material that can be efficiently utilised for organic pollutants removal during waste-water treatment and other purification processes. They claimed ZnO is a cost effective, non-toxic and more efficient photocatalyst than TiO_2_ as it absorbs a greater fraction of the solar spectrum. Moreover, they classified nZnO into several categories [48]. Generally, nanomaterials are classified (on the basis of their structure and shape) from 0 to 3 in dimensions. Spherical nanoparticles are zero dimensional (0D); nanorods, nanowires, nanotubes and nanobelts are one dimensional (1D); nanosheets, nanolayers, nanofilms, graphene and nanocoatings are two dimensional (2D); porous nanostructures (nanoflowers) are three dimensional (3D) and further subdivision gives us quantum dot arrays, respectively [49]. Figure 4 illustrates the field emission scanning electron microscopy (FESEM) images with different morphologies and different dimensions of nZnO collected by various researchers [50,51,52,53].

**Figure 5:**
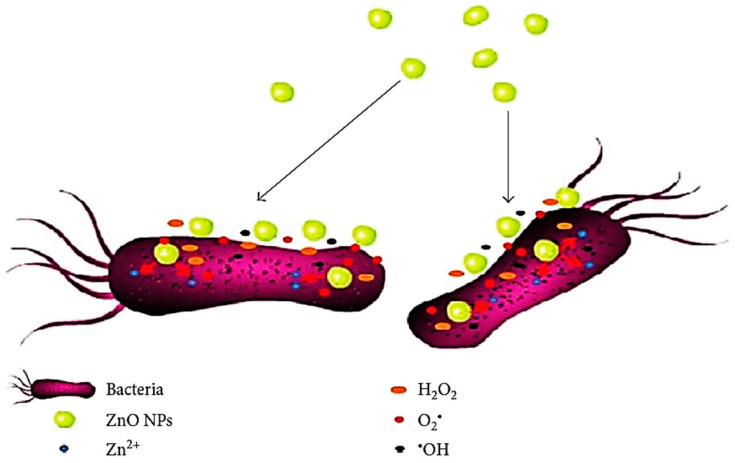
Graphical representation of antimicrobial activity of nZnO. Reprinted with permission from Reference [58]. Copyright 2018, with permission from Hindawi.

From a medical point of view, zinc is an important element that exists in tissues all through the human body. Zinc plays a vibrant role during the synthesis of proteins, nucleic acid, neurogenesis and hematopoiesis by taking part in metabolism as zinc is the major component of different enzymatic systems [54]. nZnO has been used in food additives as it is easily absorbed or digested by the human body. The US Food and Drug Administration (FDA) graded ZnO as a safe material [55]. Due to availability, non-toxicity and being less expensive as compared to other metal oxides, nZnO has been given preferences in anti-cancer, bioimaging and drug-delivery applications [18,56,57]. Jiang et al. reviewed latest developments in the fabrication of nZnO for biomedical applications. They defined some valuable characteristics, i.e., biocompatibility, less toxicity, easy access and economic efficiency that enable nZnO to be a suitable and competent material for biomedical applications. They explained that crystal growth is an important factor for biomedical application and in the case of the sol-gel method, the crystal growth of nZnO is much more significant as compared to other methods, i.e., chemical precipitation, pyrolytic process, biological process and solution free method. Their results show that nZnO (due to larger surface area) enhances the intercellular generation of reactive oxygen species (ROS) and damages the cancer cells. Moreover, they reported a potential antimicrobial effect of nZnO against a number of Gram-positive and Gram-negative pathogens. The schematic representation of antimicrobial activity of nZnO is illustrated in Figure 5 [58]. The main factor for excellent performance of nZnO is the excessive generation and induction of ROS. Moreover, the performance is based on the accumulation of nZnO in the cytoplasm of bacterial cell walls or in the outer membranes and the release of Zn^+2^ ions. This mechanism disintegrates the cell membrane and damages the membrane protein that results in killing of bacteria.

It has been revealed from the discussion that physical, mesoporous and chemical properties of nZnO are very critical and fascinating as these properties provide a theoretical description of key parameters, i.e., size, elasticity, energy distribution and thermodynamics of nZnO. Therefore, this paper summarises the important findings of research carried out in recent years on nZnO coated textiles and their enhanced photocatalytic activity.

## 2. Synthesis of nZnO and Coating Process on Textiles

In principle, there are two approaches, i.e., top–down and bottom–up, used for the synthesis of nanostructures. The first one uses grinding, cutting, slicing and milling processes to get nanostructures from bulk material. Conversely, the bottom–up approach arranges atoms and molecules either by physical methods (sonication, physical vapour deposition and thermal evaporation); chemical methods (sol-gel, hydrothermal, chemical vapour deposition, precipitation and solvothermal) or biological methods (controlled deposition and growth) to create nanostructures. The top–down approach is not suitable for photocatalytic performance as this technique causes serious damage to the crystal structure of nanomaterials, that significantly affects overall properties. Therefore, bottom–up techniques are the best to fabricate photocatalytically active nanomaterials or functional nanomaterials.

Water-based chemical methods (often called wet chemical methods) offer several benefits, i.e., low energy input, uncomplicated equipment, cheap, easy to handle reagents and being environmentally friendly [59,60]. Furthermore, they make tailoring of synthesis variables easy throughout the process that helps in gaining control over the size, shape, structure and composition of the resulting nanomaterials. Therefore, in this section, the most used chemical methods such as sol-gel, hydrothermal and coprecipitation with their influence on reaction variables that affect the crystallization kinetics, morphology, particle size distribution and facet formation of nZnO will be discussed in detail.

**Figure 6:**
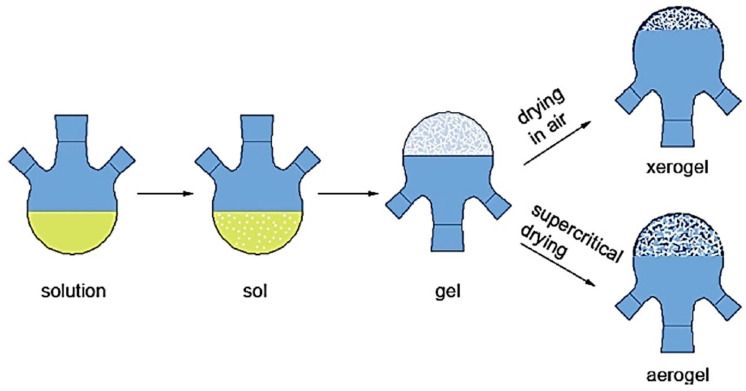
Graphical illustration of sol formation in liquid phase, infinite viscosity of gel, shrinkage of xerogel and an aerogel without shrinkage. Reprinted with permission from Reference [64]. Copyright 2012, with permission from American Chemical Society.

The sol-gel method is considered to be one of the most versatile methods to fabricate advanced and novel materials especially nanomaterials [61,62,63]. The sol-gel method initializes with hydrolyzation, polymerization and ends with condensation reactions. This method comprises the preparation of a colloidal solution (generally known as sol) that is further converted into gel. The precursors generally used for the sol-gel method are metal alkoxides and chloride salts. For nZnO, the most common precursors are zinc chloride (ZnCl_2_), zinc nitrate hexahydrate (Zn(NO_3_)_2_·6H_2_O) and zinc acetate Zn(CH_3_CO_2_)_2_. The most important variables that affect the size, shape and dimensions of nZnO in sol-gel synthesis are the concentration of solvents, nature of solvents, temperature and molar ratio. Sui and Charpentier provided a detail explanation about sol-gel nano synthesis mechanism of metal oxides for supercritical fluid applications. They explained the benefits of supercritical fluids and supercritical drying in the synthesis of solid products. It is already known that at ambient conditions, some inevitable shrinkage of the solid leads to the collapse of the microstructure that results in low specific surface area. The formation of sols in the liquid phase gets an infinite level of viscosity when converted into a gel results in the formation of xerogel and aerogel with ambient drying and supercritical drying respectively. The results explain the overall chemistry of metal oxide-based nanomaterials synthesised by the sol-gel process and suggest the use of supercritical fluids i.e., H_2_O, CO_2_ and organic solvents in nanofabrication. The graphical representation of the mechanism of the sol-gel synthesis is illustrated in Figure 6 [64].

**Figure 7:**
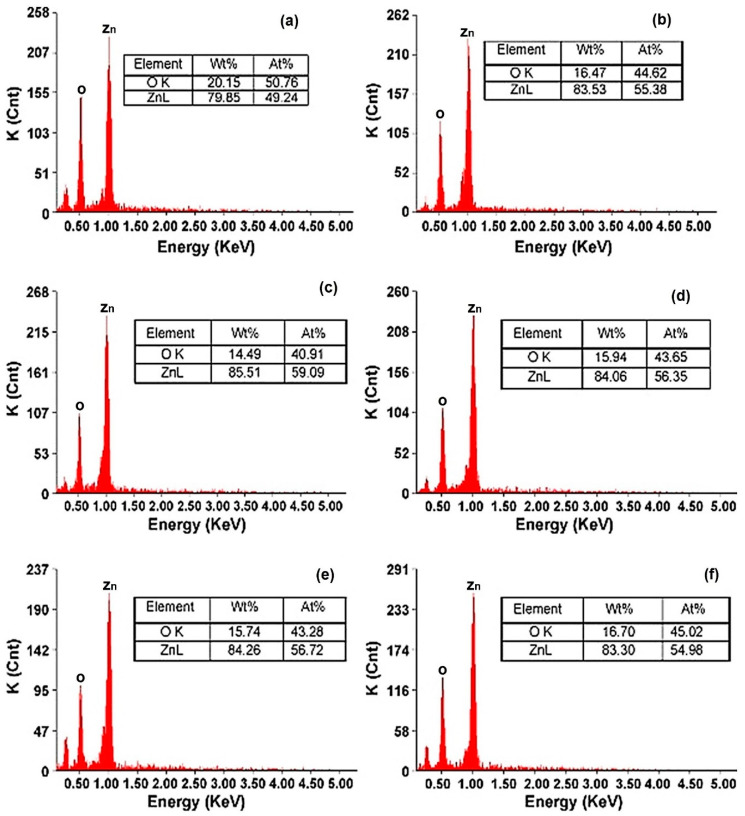
(**a**–**f**) Elemental detection spectroscopy (EDX) analysis of sol-gel synthesised nZnO at varies pH from 6 to 11, respectively. Reprinted with permission from Reference [65]. Copyright 2010, with permission from Elsevier.

**Figure 8:**
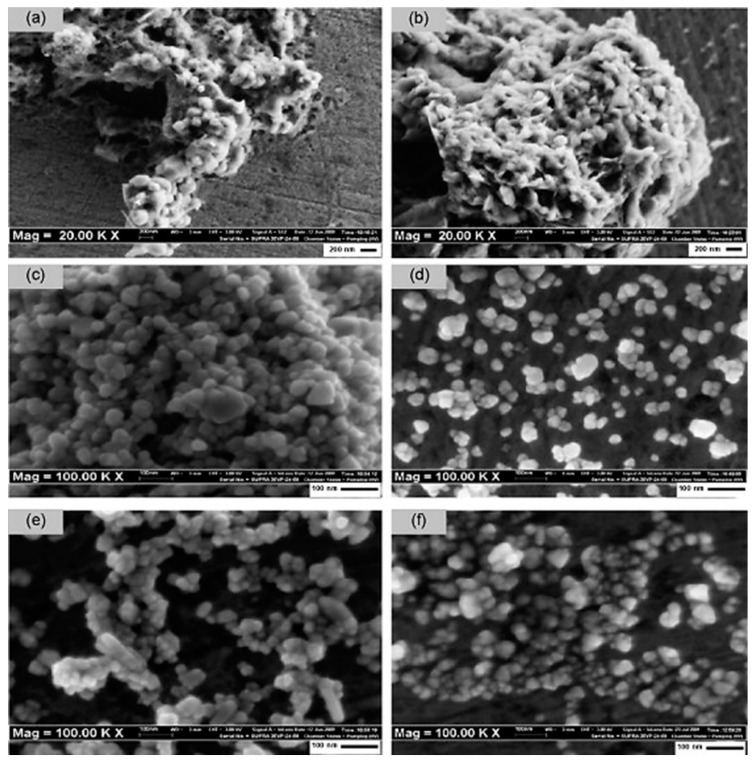
(**a**–**f**) FESEM analysis of sol-gel synthesised nZnO at varies pH from 6 to 11, respectively. Reprinted with permission from Reference [65]. Copyright 2010, with permission from Elsevier.

Alias et al. reported sol-gel synthesis of ZnO NPs under different pH conditions. The resulting nano powders showed agglomeration at pH 6 to 7 (acidic and neutral conditions). However, alkaline pH conditions (pH = 9) were more favourable in obtaining nZnO powders. The sizes of the resulting nanoparticles were lower in acidic medium. However, the optical properties were better in alkaline conditions. The sizes were significantly uniform at pH 9 to 11 and the size range was 37 to 50 nm at pH 9 and 11, respectively. Chemical composition of as synthesised nZnO explained the existence of methanol solvent near the x-axis origin. The results of energy dispersive X-ray (EDX) analysis confirm the Zn and O elemental peak for all pH levels as illustrated in Figure 7. The results also explain the growth mechanism of nZnO in a precise manner. For sol-gel synthesis, hydrolysis and polycondensation (nucleation) are the primary steps to prepare particles, while for growth of nZnO with high crystallinity, a sufficient amount of OH^¯^ ions are necessary. The results of FESEM for surface topography, structural and morphological analysis at different pH conditions are presented in Figure 8 [65]. Gupta et al. achieved a uniform nZnO coating on cotton by the sol-gel method and fabricated smart wearable electromagnetic interference (EMI) shielding electrically conductive textiles that protect the human body from electromagnetic pollution [66].

Hydrothermal is another widely used wet chemical method that uses autoclaves under controlled conditions. In autoclaves, the temperature rises above 100 °C and reaches to saturated vapor pressure. This method is generally followed in order to achieve small size particles. Commonly used precursors for hydrothermal synthesis are zinc nitrate hexahydrate and zinc sulphate heptahydrate (ZnSO_4_·7H_2_O). The variables that affect the size, shape and dimensions of nZnO in hydrothermal synthesis are pH, calcination temperature and heating time. In an experimental study, Gong et al. incorporated hydrothermally synthesised nZnO over the optical fibre surface and evaluated the growth mechanism. The results reveal that an augmentation in preferred growth and less oxygen vacancy are mainly due to higher UV irradiation power [67]. In a recent study, Koutavarapu et al. reported hydrothermally synthesised ZnO nanosheets based hybrid nanoribbons. Photocatalytic performance was evaluated against tetracycline, a toxic organic pollutant. The results show 98% photodegradation of tetracycline within 90 min [68].

Precipitation and coprecipitation methods are also very renowned to fabricate nZnO and have gained attention in recent years. These methods involve a reaction of zinc salts (zinc precursors normally ZnSO_4_·7H_2_O, Zn(CH_3_CO_2_)_2_, Zn(NO_3_)_2_·6H_2_O and ZnCl_2_) with alkaline solutions i.e., NaOH, KOH, LiOH and NH_4_OH. The reaction starts between Zn^2+^ and OH¯ that follows aggregation and a stable colloidal suspension of nZnO is formed in the presence of alcohol. nZnO with different morphologies are obtained by controlling the typical variables, i.e., pH of the solution, concentration of the solution, type of precursor, calcination temperature and type of alkali, of these processes. By using the precipitation method, Shetti et al. reported the synthesis of nZnO taking 0.2 M Zn(NO_3_)_2_·6H_2_O and 0.4 M KOH and further applied it on carbon—nZnO coated carbon electrode designed to detect Molinate, a thiocarbamate herbicide, by cyclic and voltametric methods [69].

**Figure 9:**
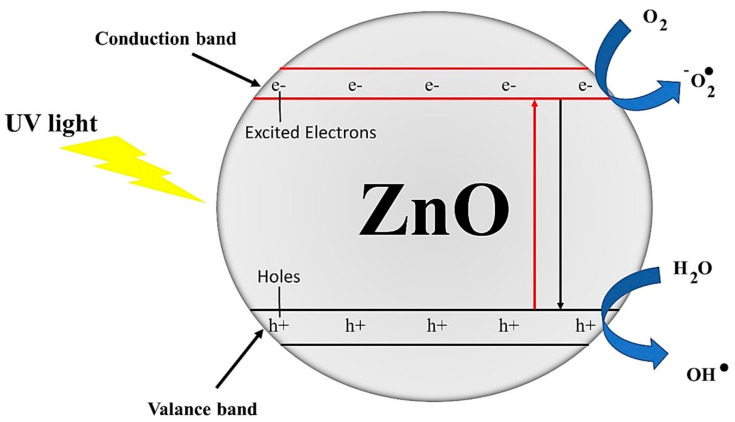
A typical mechanism of photocatalytic activity of pure nZnO. Reprinted with permission from Reference [70]. Copyright 2021, with permission from Elsevier.

**Figure 10:**
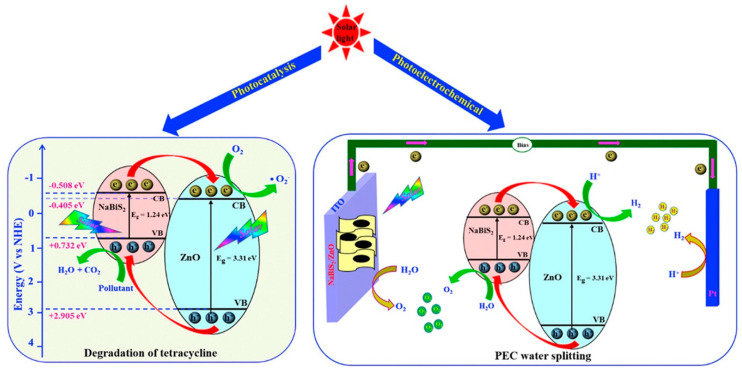
A comparative analysis of photocatalytic activity of doped nZnO for the photodegradation of organic pollutant (tetracycline) and for photocatalytic water splitting. Reprinted with permission from Reference [68]. Copyright 2021, with permission from Elsevier.

The wide band gap of ZnO allows this material to function only in the UV range. In addition, surface oxygen vacancy and a higher recombination rate of photo generated charge carriers decreases the photocatalytic efficiency of ZnO. The defects in ZnO structure defined by nonbonding electrons in polarization, band gap and elastic modulus. The crystal size is associated with annealing temperature. The surface defects, interatomic and atomic interactions and variations in electronic distribution are the dominant parameters in determining the photocatalytic performance. Doping is an excellent method to overcome these issues by tailoring the optical properties of nZnO. Doping comprises the insertion of a specific element (ions) into the crystal lattice of ZnO that modifies and controls the band gap of ZnO with a direct consequence on the photocatalytic activity of ZnO. The physicochemical properties of doped ZnO significantly depend on dopant type. For nZnO, commonly used doping agents are cerium, titanium dioxide, calcium, boron, cobalt and magnesium. Doped ZnO is generally considered as a hybrid multifunctional metal oxide and is significantly used for photocatalytic, sensing, energy and biomedical applications. In a recent study, Kim and Yong fabricated boron doped ZnO and reported a significantly higher photocatalytic hydrogen production. They observed that boron doped ZnO illustrates type 2 alignment of band structures whereas undoped ZnO exhibits z-scheme band. Due to this dramatic change in band structures, boron-doped ZnO shows a 2.9-times higher H_2_ production rate than undoped ZnO [31]. The reason for excellent photocatalytic activity of nZnO is the formation of ROS. Photocatalytic reaction initializes when a light beam strikes the surface of nZnO. The formation of excess numbers of ROS (due to larger surface area of nanostructures) with their strong power of decomposition degrade organic pollutant that cause staining and health issues [70]. The photocatalytic activity of nZnO in bare and doped forms for photodegradation of organic pollutants and solar driven water splitting is graphically illustrated in Figure 9 and Figure 10, respectively.

Table 1 summarises a brief overview of other synthesis methods used in the fabrication of nZnO.

## 3. Photocatalytic Applications

**Figure 11:**
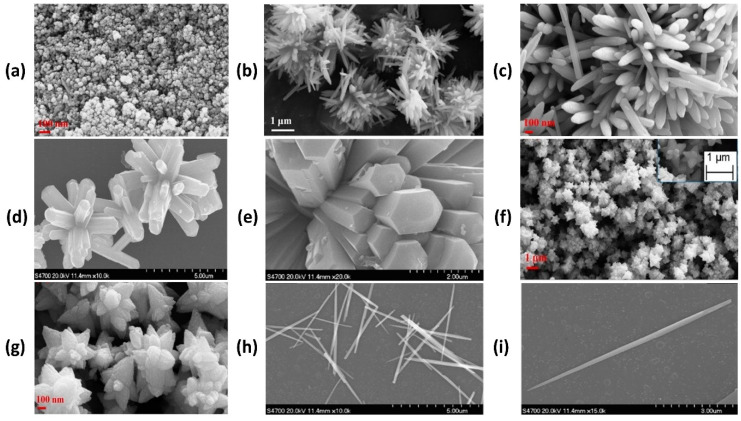
Illustration of different morphologies of nZnO taken by FESEM analysis: (**a**) ZnO nanoparticles; (**b**–**e**) ZnO nanorods; (**f**,**g**) ZnO nanoflowers; and (**h**,**i**) ZnO nanoneedles. Reprinted with permission from References [89,90]. Copyright 2010, 2013 respectively, with permission from Elsevier.

For photocatalytic applications, higher photocatalytic activity and optical properties are key features that enable nZnO a good choice to be utilised as a potential (photocatalyst) material. nZnO is an extensively used material as a photocatalyst in the photodegradation of various organic pollutants. Many researchers have utilised nZnO in the textile sector. Ghayempour and Montazer reported a coating mechanism of nZnO on a cotton surface with the help of tragacanth gum—a natural biopolymer—and ultrasonic energy. The results reveal a wurtzite structure with star like shape of as synthesised nZnO that shows good to excellent photocatalytic efficiency against the photodegradation of methylene blue (MB) [81]. In another study, Chakrabarti and Banerjee coated sonochemically synthesised nZnO on cotton fabric by padding to achieve multifunctional characteristics. The results explain that developed samples show significantly enhanced photocatalytic properties and degrade 69% trypan blue, a direct azo dye, under solar self-cleaning [82]. In the photodegradation of organic pollutants, OH^●^ radicals take part in a redox reaction and convert the long chain molecules into H_2_O and CO_2_. In a recent study, Noman and Petru reported a sonochemical coating of nZnO on cotton fabric under controlled conditions and compared the comfort performance against TiO_2_. In an in-situ coating, ZnCl_2_ was used as a precursor and an average particle size of 30 nm was successfully achieved. The results depict that nZnO coated fabric exhibits higher properties than TiO_2_ coated samples [83]. Both doped and undoped ZnO has been used for photocatalytic applications and preference is given to the type of application, i.e., for photodegradation of organic pollutants, mostly pure nZnO is used while for photocatalytic water splitting, energy and sensing applications, doped nZnO is preferably used. The previous literature elucidates that pure nZnO works better in the degradation applications and doped nZnO in other applications. Nair et al. worked with undoped and cobalt-doped nZnO and performed photocatalytic experiments against MB solution and reported that photocatalytic efficiency of pure ZnO is significantly higher than cobalt doped ZnO. The reasons for higher photocatalytic activity are zinc vacancies and interstitial oxygen atoms from acceptor states and oxygen vacancies and interstitial zinc atom from donor states [84]. In another study, Kaur and Singhal synthesised various metal doped nZnO and evaluated photocatalytic performance against methyl orange (MO). The results show that MO degradation follows first order reaction kinetics and undoped nZnO performs much better than metal-doped ZnO [85]. Photocatalysis is a dynamic process that triggers a series of redox (reduction and oxidation) reactions and convert long chain molecules into less toxic materials, i.e., CO_2_ and H_2_O. The primary oxidizing species that work during photocatalysis are superoxide anions and hydroxyl radicals. It is observed from all above discussed literature that different morphologies of nZnO perform differently during photocatalytic applications and significantly affect the photocatalytic performance. One dimensional nanorods, nanoneedles and nanowires provide better results during photocatalytic applications as compared to nanoparticles because they provide more surface area than nanoparticles. FESEM images of nZnO with various morphologies like nanoparticles, nanorods, nanoflowers and nanoneedles are provided in Figure 11.

For hydrogen generation, electrochemical and biosensors, doped nZnO has opened a new research area because doping of suitable element enhances the efficiency of nZnO to a significant level for these applications. In a recent study, Bukkitgar et al. reported excellent electrocatalytic sensing performance of Mg-doped ZnO nanoflakes for an anti-inflammatory drug, mefenamic acid. The results reveal that pH and selection of dopant are important variables to control the sensing performance of doped nZnO as well as to develop durable and highly efficient sensors. Dopant selection significantly affects the electrochemical detection limit and offers better results under low detection limit [86]. In their previous study, barium was loaded as a dopant and Ba doped ZnO was used to detect mefenamic acid under cyclic voltammogram. The results show good recovery values and suggest the developed sensors for practical use [87]. However, the role of bare/undoped metal oxide semiconductors especially ZnO for solar photocatalytic applications is still undeniable. In a recent review article, Karthikeyan et al. elucidated the pivotal aspects of utilising different types of metal oxide semiconductors as a photocatalyst in hydrogen production, dye sensitised solar cells, energy storage batteries, water splitting, electrodes and sensors. They covered zinc oxide, titanium dioxide, copper oxide, tungsten oxide, tin oxide and their nanostructures and specified the vibrant role of nano dimensions for solar photocatalytic applications [88].

## 4. Future Direction

The contributions in this review paper can lead new comers to new lines of inquiry about the role of photogenerated charge carriers (ROS, radical scavengers) on the surface morphology of polymeric fibrous material and on photocatalytic applications. The discussed literature clarifies that synthesis methods and the selection of precursors are very important in order to get better results for photocatalytic applications. The latest progress and discussion particularly in these areas have been elucidated in this thematic issue in order to understand the variables and their combined effect on better photocatalytic activity. Zinc oxide nanostructures as a photocatalyst attract enormous attention due to their potentials of harnessing solar energy directly for the production of hydrogen and solar fuels, and degrade toxic pollutants as well. However, the efficiency of nZnO is reduced due to faster electron-hole recombination rate and low light consumption. The fabrication of high-quality photocatalysts on largescale for real time applications is still a challenge. Advancement is also necessary for the fabrication of an efficient, sustainable, cost-effective and facile photocatalyst. Therefore, there is still a paucity to go deeper and investigate other dimensions that have emerged in light of the findings presented here.

## 5. Summary

Zinc oxide nanostructures have high specific surface area, optical properties and flexibility that elucidate their potential for excellent photocatalytic applications especially photodegradation of organic pollutants, self-cleaning and antimicrobial efficiency. Higher photocatalytic activity significantly depends on synthesis routes, precursors type, selected variables and dopant type. Doped nZnO shows excellent results for water splitting, H_2_ production, sensing and electrochemical detection of various materials. It was observed that doping shifts the bandgap and provides more radical scavengers, i.e., OH^●^ radicals. The discussed literature elucidates that the synthesis methods control the crystallinity, dimensional stability, particle size and overall photocatalytic activity of nZnO. Moreover, an extensive investigation of photocatalytic activity and the role of photogenerated charge carriers (ROS) provide a comprehensive knowledge of reaction kinetics and crystal structure for next generation materials and devices.

## Figures and Tables

**Table 1:** Overview of some synthesis methods for nZnO.

Method	Used Precursors	Conditions	Structure; Size	References
Ultrasonics	Zn(NO_3_)_2_·6H_2_O, C_6_H_8_O_7_·H_2_O	Sonication for 0.5–2 h, room temperature	Hexagonal, spherical particles	[71]
Chemical bath deposition (CBD)	Zn(CH_3_CO_2_)_2_·2H_2_O, C_2_H_4_(NH_2_)_2_	80 °C temperature, magnetic stirring up to 3 h	Core shell rod like structure, size 500 nm	[72]
Sol-gel	Zn(CH_3_CO_2_)_2_, C_2_H_5_OH, (C_6_H_9_NO)n	100 °C temperature, stirring time 2 h, calcination at 300 °C	Size 140 nm	[73]
Sol-gel	Zn(CH_3_CO_2_)_2_·2H_2_O, C_2_H_5_OH, NaOH	60 °C temperature, time 17 h, annealing at 200 °C	Hexagonal wurtzite, spherical, size 22 nm	[74]
Co-precipitation	Zn(CH_3_CO_2_)_2_·2H_2_O, NaOH, C_2_H_5_OH, NH_4_OH	Agitation time 1 h, annealing at 60 °C, for 6 h	Nanorods, wurtzite crystal structure	[75]
Successive ionic layer adsorption and reaction (SILAR)	ZnSO_4_⋅7H_2_O, NH_4_OH, CuSO_4_·5H_2_O	Dipping cycles 30, annealing 500 °C for 2 h	Hexagonal wurtzite, spherical, size 34 nm	[76]
Hydrothermal	Zn(NO_3_)_2_·6H_2_O, NaOH, Zn(CH_3_CO_2_)_2_·2H_2_O, C_2_H_5_OH,	Heating 50 °C, stirring 30 min, oven drying 150 °C for 6 h	Nanorods, wurtzite crystal structure	[77]
Two step sonication modified sol-gel	Zn(CH_3_CO_2_)_2_·2H_2_O, NH_4_OH, KOH, Tetraethyl orthosilicate (TEOS)	60 °C temperature, time 1.5 h, sonication time 1 h	Hexagonal wurtzite, spherical nanoparticles	[78]
Biological	Tabernaemontana divaricata leaves extract, Zn(NO_3_)_2_·6H_2_O	80 °C temperature, continuous stirring, drying at 450 °C for 2 h	Hexagonal wurtzite, spherical, size 20–50 nm	[79]
	Abutilon indicum leaves extract, Zn(NO_3_)_2_·6H_2_O	Temperature of furnace at 200 °C for 3 min., calcination for 2 h	Hexagonal wurtzite, spheroid rod like shape, size 16–20 nm, band gap 3.36 eV.	[80]
